# "The Hospital" Nursing Mirror

**Published:** 1899-06-24

**Authors:** 


### "The Hospital" Nursing Mirror.

Being the Nursing Section of "The Hospital."

[Contributions for this Section of "The Hospital" should be addressed to the Editor, The Hospital, 28 & 29, Southampton Street, Strand, London, W.C., and should have the word "Nursing" plainly written in left-hand top corner of the envelope.]

### Notes on News from the Nursing World.

### NURSING IN PRISONS.—QUESTIONS IN PARLIAMENT.

A very important series of questions will be put by Mr. Parker Smith on Monday in the House of Commons to the Lord Advocate. They arise out of the personal experiences of the member for the Partick division of Lanarkshire. It appeared that he recently paid a casual visit to Duke Street Prison, Glasgow, and found a sick untried female prisoner being nursed by two convicted prisoners. The allegations which he is to bring before the notice of Parliament are as follows: On October 14th last, he states, Mary Carroll, being under treatment in the prison for a gangrened foot, was left alone in an ordinary cell; she was taken seriously ill in the night, and the nurse-warder being summoned, she was removed to an association cell already occupied by a sick prisoner under the charge of two other prisoners; there she was left in charge of the two prisoners, who about an hour afterwards rang the bell and reported that she had a bad turn, and, on the arrival of the nurse-warder, she was found to be dead. Mr. Parker-Smith accordingly wishes to learn from the Lord Advocate "what was the report of the doctor upon the case; what were the qualifications of the nurse-warder to judge as to the condition of the patient; whether any steps have been taken to ensure that prisoners seized with serious illness shall receive attention; and whether he will provide that in so large a prison there shall be a proper hospital where all the sick can be placed, and a properly trained nurse in constant attendance." Unless the answer to these interrogations is satisfactory, the matter will certainly have to be pursued further.

### UNDER THE GENEVA FLAG.

If the only result of the Peace Conference at the Hague is to put into force the proposals for observing the provisions of the Geneva Convention in future naval battles, it will not have been held in vain. In the probable event of the scheme of the Conference being carried out, the hospital ships in naval battles will henceforth be painted a distinctive colour and fly the Geneva flag; they will be respected during hostilities; they will take help to the wounded, sick, and shipwrecked without distinction of nationality; and they will not be liable to capture or their occupants to be made prisoners of war. It is obvious that the adoption of these humane and admirable proposals would have the effect of ensuring for the sick and wounded in naval battles much better attention than it has been possible to afford in the past; while as a provision for the security of the nurses in the hospital ships they are absolutely indispensable.

### THE CATHEDRAL NURSE AND LOAN SOCIETY.

At the annual meeting of this society in Newcastle-upon-Tyne a highly satisfactory report was presented by the committee. The record of work done by the nurses was as follows: 1,823 cases visited, 16,176 visits paid, 186 times night nurses employed. The parishes of Benwell, Gosforth, and Jarrow are still worked by one of the cathedral nurses, by their own payments; and the financial statement shows that the income for the year exceeded the expenditure by the substantial sum of £214. The Vicar of Newcastle, Canon Gough, in moving the adoption of the report, speaking from more than 50 years' experience of parochial work, bore testimony to the usefulness of the society, and Dr. Paget, who seconded, said he had heard from patients most glowing accounts of the way in which the nurses looked after them in their own homes. Dr. Hawthorn, in proposing a resolution recognising the value of the society's work, observed that he had had occasion to send at most inconvenient times to Miss Colman and her staff, but they had invariably responded most promptly and efficiently.

### A PARSIMONIOUS BOARD OF GUARDIANS.

Notwithstanding the arguments and facts urged in favour of a subscription to the funds of the Newtown Nursing Association, the Caersws Board of Guardians have declined to vote a £5 note for the purpose. The excuse for the parsimony is that well-to-do persons, and not the poor, benefit by the services of the nurse who resides in Newtown. It was, however, pointed out at the meeting of Guardians that the well-to-do people who occasionally employ the nurse pay her for her attendance, but that most of her time is occupied in visiting the poor. Even on grounds of economy it was contended that the Nursing Association is entitled to support, as before the nurse came the Guardians incurred a large expenditure in paying people to attend outdoor cases, while since her advent that system has been done away with, and the money of the ratepayers has been saved. But the contention fell on deaf ears, and the Caersws Board will have neither part nor lot in the movement which has been so warmly supported in Merionethshire.

### WORKHOUSE NURSING IN EAST ANGLIA.

The question of workhouse nursing is exciting the keenest interest in East Anglia, and the *Eastern Daily Press* demands the prompt intervention of the Local Government Board. It is quite clear that the Poor Law guardians are altogether unable to cope with the difficulty of obtaining trained nurses for the paupers. At a recent meeting of the Board of Guardians of a district in Norfolk a nurse who had sent in her resignation was called to place before the Board her reason for having taken this step. It was the usual one of discomfort and general disgust at most of the workhouse arrangements. The chairman said "he really did not know what to do; nurse after nurse resigned, till it seemed hopeless to imagine they could ever be settled again." This is only one of many examples which come to notice, almost daily. As the *Norfolk Standard* says, the training of the workhouse nurse is not the only difficulty :—

"There is the question of her control, of her accommodation at the infirmary, of her hours of work. The student of the reports of the various guardians' meetings in city and county cannot fail to be struck with the fact that the workhouse nurse is continually on the move. She is appointed to-day and gone to-morrow. What is the reason? In nine cases out of ten because her comfort and convenience is the last thing thought of. Her control is provided for, and under that, with no sympathy or kindly consideration in the dreariest of tasks, she chafes till she seeks relief by throwing up her post. Meanwhile, the old pauper nurses, the wretched old crones we knew so well when we wept over the woes of little Oliver Twist, are still, to a large extent, in possession."

Of course, the worst of it is that all the time the unfortunate paupers, whose infirmities are always specially hard to bear, suffer from the inability of the local authorities to grapple with the new situation.

### A PLEASANT CHANGE FOR NURSES.

There are hundreds of carriages in London at the present moment that are not in constant use. We wonder how many of their owners have ever thought of one way in which they might be turned to admirable account—employed to afford pleasure without expense. The other day we saw three nurses enjoying a drive in a carriage with a pair of horses. But the spectacle is a rare one. Perhaps the result of mentioning it may be to secure an hour's fresh air in the most delightful manner for some of the nurses whose opportunities of change are few, and who, when they are able to go out, are often far too tired to walk.

### THE NEW DEPARTMENTS OF THE YOUNG WOMEN'S CHRISTIAN ASSOCIATION.

A new feature of the varied work of the Young Women's Christian Association deserves mention. The committee have started agencies for the help of deaf and blind girls to whom the advantages of membership in the association are now secured by means specially adapted to their needs, such as, for instance, the printing in Braille type of the monthly letter. From the report of the association for 1898 it appears that several hundred girls in business have been lodged in their 26 homes in different parts of London, 1,026 have been helped to situations by its employment agency, and 340 women and girls have received assistance in time of need from its convalescent, sick aid, and holiday department. During last summer 5,000 girls availed themselves of the free invitations to country gardens near London, arranged by the same department, while at the holiday home of the association, Peace Cottage, near Rickmansworth, 300 young women have taken rest and change for longer or shorter periods.

### THE HUMOURS OF DISTRICT NURSING.

Although district nursing is now so general and so rapidly growing into favour, even in our small country districts, it is quite surprising to find how very ignorant some people are respecting the nurses and their work. A correspondent says she has been much amused by the experiences of a district nurse, who has only a few months since taken up quite new work in a Yorkshire village. Although her district contains about 4,000 inhabitants she has come across many who have never even seen a trained nurse, and some of their remarks are very funny. For some days one old lady of about seventy years of age refused to let the nurse wash her and make her bed. She had never seen a nurse in her life, and thought the white bonnet strings and linen apron and sleeves quite too grand for a "working woman," and she should sit down and talk a "bit." The mother of a girl in the last stage of phthisis was so taken up in gazing at the nurse that she could not listen to her instructions as how to make egg-flip, and after the nurse had told her three times the girl burst out with "Eh, my, but you have some grand teeth; a'rt pot 'uns." The nurse's instructions were useless. In another case a little girl, about five years old, for weeks imagined the new nurse was the Queen. As soon as the nurse turned down into her street, the child ran indoors crying out "Grannie, come quick, there's the Queen coming, and she has got on such a beautiful big white bow." A little boy was quite certain for a long time that the nurse was the doctor's wife, and when told she was not exclaimed, "Well, she cures us, and so does doctor; why is she not his wife?"

### THE EXPEDITION TO THE WEST COAST OF AFRICA.

Major Ross, whose lecture at Liverpool on "Nursing in the Tropics" was referred to last week, starts in August on an expedition which requires as much courage as going to the front in time of war. The members of the expedition will purposely reach Sierra Leone in the height of the malarial season, in order to pursue their researches under the most favourable conditions. Whether or not Major Ross succeeds in proving his theory that malaria is caused by the bites of a certain species of mosquito, his investigations cannot fail to be of great value, and their progress will be watched with the keenest interest in the nursing world.

### CYCLISTS AND NURSING.

From far Lockerbie we have an interesting report of a very gratifying instance of the popularity of the district nurses. Under the auspices of the Lockerbie Cycling Club there was a few days ago a fancy dress parade of a very successful character, in which a large number of cyclists in all possible costumes—from that of Mephistopheles to that of a clown—took part. As an indication of the interest manifested in the proceedings, it may be mentioned that, by the desire of the magistrates and commissioners, all places of business in the town were closed early in the evening. This was done in order to swell the attendance at the parade, and especially the amount of the collection, the latter being given to the Lockerbie Nursing Association. After payment of all expenses, including prizes to the boys and girls who collected the most money at the parade, the committee were able to hand over to the association the sum of £15 10s., a very welcome contribution from the cycling club.

### MISS SHARMAN'S ORPHAN HOME FOR GIRLS.

The most interesting feature of the annual meeting at the Mansion House on Friday in connection with Miss Sharman's Orphan Homes was the speech of Miss Sharman herself. In a brief account of her work she stated that she opened her first home in May, 1867, for ten children. Since then a new home has been built in Austral Street, West Square, at a cost of over £21,500, and country branches added at Gravesend, Tunbridge Wells, and Hastings. The number of girls has increased to 323, and a total of 1,427 children have been received and cared for during the past 32 years. There is a branch at Stockwell for children of superior birth, and a detached infirmary belonging to the home. All the homes are freehold, and the property is unencumbered by debt or mortgage. The children are received from babyhood, and each elder girl is entrusted with the care of a younger one; the school work is carried on on Board School lines, and careful training in needlework and domestic work is given. No servants are kept, the elder girls doing the work under the direction of the matrons, and thus preparing for service. Orphanhood and destitution are the qualifications for admittance, the average cost of each child's support in the London home being £15 per annum. There are no management expenses, the services of superintendent, doctors, architects, &c., being given gratuitously, and no canvassing or begging appeals are ever issued. Yet somehow the funds have been found to carry on the work, although, as Miss Sharman explained, no brick has ever been laid until there was money to pay for it. The report states that a good bill of health can be shown, and the death-rate has been considerably below 1 per cent., notwithstanding that very young and very delicate children are admitted.

### A LAMENT FROM BRITISH COLUMBIA.

The splendid snow-crowned mountains round me
Lift their heads,
The snow-fed mighty rivers thunder down
Their rocky beds,
Vast forests clothe the lonely mountains'
Lower slopes,
And I, an unknown nurse among these
Scenes, had hopes
Of comfort bringing when disease was
Near, or death,
Or skilled attendance when new life it-
Self drew breath;
Of nursing all I could, then setting
Forth for home,
For England is my magnet whereso-
E'er I roam.
Alas! one little barrier stops this
Simple plan—
What often does prevent the best laid
Plans of man;
A simple lack of dollars for the
Rail and wave,
Howe'er so hard I try to work and
"Skimp" and save.
Meanwhile I struggle on through much that
Is adverse,
To prove the worth and honour of an
English nurse;
Content to toil where all is strange and
Do my best,
Still hoping 'fore I'm old in England
I may rest.
Till then, I dream when twilight gray doth
Dim the day
Of all the faces dear that I recall
So far away;
Of hospital and matron, sisters,
Nurses bright,
Of all my life surroundings where all was
Life and light. H.

### NURSING IN GLASGOW.

From the fourth annual report of the committee appointed to provide and maintain resident trained nurses for the district of Maryhill we learn that it has become necessary to take a house in lieu of apartments. The number of cases attended in 1898 by the ten nurses engaged was 238. A satisfactory feature of the work is that, whereas in the first year the number of subscribers to the scheme was only 459, it is now 1,336, an increase of nearly 200 per cent.

### THE LONDON SCHOOL NURSES' SOCIETY.

As will be seen from our report of the first annual meeting this society has, in 12 months, amply justified its existence. By the help of kind friends it has met its liabilities, and has kept three nurses going. But the work in London requires ten. This means that an income of £540 is needed. Sir Sydney Waterlow is so well pleased with the work of the society that he has doubled his subscription. We hope that other subscribers will follow this admirable example, and that the balance will be provided by people who have been waiting to learn the record of work done.

### THE INTERNATIONAL CONGRESS OF WOMEN.

We learn that the Hon. Sydney Holland is inviting nurses attending the International Congress of Women to visit the London Hospital on Thursday, June 29th, at four p.m., when the hospital will be inspected, and Mr. Holland will explain the system of training and nursing. Miss Lückes will afterwards entertain the guests in the garden at tea. Nurses accepting this invitation should write to Miss Dallas, 2, Bryanston Square, before Monday next. Miss Stewart asks nurses to tea at St. Bartholomew's on June 28th, at four p.m. The National Association for Promoting the Welfare of the Feeble-Minded asks those interested in the training of mentally deficient children to St. Saviour's Home, Hendon, on March 27th. Tea at half-past four. The women's clubs and settlements are all extending hospitality to members of Congress.

### SHORT ITEMS.

On Thursday afternoon, at half-past three, the Duchess of York will open the garden fête at Kidbrook Lodge, Blackheath, in aid of the Marchioness of Dufferin's Fund for supplying female medical aid to the women of India.—The marriage of Mr. L. V. Harcourt and Miss Burns will take place at St. Margaret's, Westminster, on Saturday, July 1st, at a quarter past two.—On Saturday, July 1st, the Countess of Portsmouth will reopen the children's ward of the Tottenham Hospital. There will be a garden party in the grounds, and Lady Portsmouth will receive purses from a number of children towards the maintenance fund of the Hospital.—The Princess Louise will distribute the badges and certificates to Queen's Nurses at Kensington Palace, on Wednesday, July 5th, at half-past three p.m.—The new buildings which have recently been completed at the London Hospital Medical College will be opened on Tuesday, July 18th, by Lord Knutsford. The opening will be followed by the distribution of prizes to the students and nursing probationers, in the Library of the Medical College, by Lord and Lady Knutsford.—The last of the series of three concerts in aid of the funds of University College Hospital was held in the Botanical Theatre, University College, on Friday afternoon, before a crowded and fashionable audience.—No less than 36 hospitals in Dublin will derive a benefit from the property bequeathed by Mr. James Weir to the charities in the Irish capital. The amount divided is £100,800.—On Tuesday it was decided at a large meeting, convened by the Duke of Portland, that a sanatorium for the open-air treatment for consumptives for the county and city of Nottingham should be established.

### Gynæcological Nursing.

By G. A. Hawkins-Ambler, F.R.C.S., Surgeon to the Samaritan Free Hospital for Women; Assistant Surgeon to the Stanley Hospital, Liverpool.

The Qualifications of the Gynæcological Nurse.—(*Continued.*)

As regards discipline, it is well to remember that when on duty you must always wear the badge of duty. An untidy appearance, a disregard of uniform, a slipshod method of doing your work are as bad for you as for the patient. You must always be neat and clean, be of quiet habit, avoid gowns that rustle and chatelaines that clank, and let your manners in the homes into which you are introduced make you respected—not for a vulgar insistence on imaginary rights, and a demand for the recognition of fancied importance, but by such a bearing as will insure you the respect always given to professional skill and consideration for the feelings of others, who are probably distracted by the distress and confusion that reign in any household where sickness has come. If you have any real dignity it will take care of itself. If you have none, no amount of airs or rudeness of manner will obtain for you the consideration which you naturally desire.

Examination of Patients.

When a patient is to be examined it may be done while she lies in bed, or at the rooms of the surgeon in attendance. If the former, the nurse, if she have notice, and if there is no order to the contrary, will give the patient a mild aperient the day before the examination is to take place. A few capsules of castor oil or a teaspoonful of liquorice powder the night before, followed by an enema of soap and water the morning of the doctor's visit, in time for it to act before his arrival, will much facilitate matters. The bowel will be emptied, and there will be less difficulty in examining the lower parts of the pelvis, which with women who are habitually careless about the calls of nature is sometimes filled with loaded intestine. It is sometimes difficult for the doctor to distinguish between growths, organs, and masses of hardened fæces in the lower bowel. The enema settles this question, and it also permits an examination by way of the bowel itself, which is one of the most valuable methods at the disposal of the gynæcologist. The nurse, then, will have taken care that the patient has an unloaded rectum. She will also be wise to arrange that the patient has been without food for three or four hours before the examination is to take place. If the visit be a morning one, a cup of Bovril taken early will help to sustain the patient, and still leave her with a comparatively empty stomach. Then, if it be necessary to administer an anæsthetic, there will be no difficulty on that score, and no irritating postponement of a duty which is at the best an ordeal for a woman to face, and which when complicated by loaded intestines, &c., is at times very painful and always unsatisfactory.

These preliminaries settled, and the patient wearing a pair of woollen drawers and vest, which protect her from cold while not interfering with the manipulations of the surgeon, she is ready for examination. But the nurse will also have in readiness plenty of hot water, a new nail brush, or one that has been lying in a 1 in 20 solution of carbolic acid overnight, towels, and a solution of one part of corrosive sublimate in 1,000 parts of water, and some antiseptic lubricant. If the sound has to be used, and though many practitioners avoid the frequent use of this instrument, it will be well to take it for granted that it may be required in the examination, it will be well to give the patient a douche of a quart of hot corrosive sublimate lotion, 1 in 2,000. When the doctor has washed his hands and is ready, the patient is first placed on her back, close to the edge of the bed, the head slightly raised by a pillow placed under her shoulders, and her knees bent. The blankets and quilt are turned well down below the knees and the patient left covered only by a sheet, with, if the room be not over warm, a light shawl over the chest. The abdomen is exposed, and the surgeon's hands are not hampered by heavy clothing.

The doctor may complete the examination with the patient lying on her back, or he may desire to have her turned on her side. In the latter case you will draw the patient close up to the edge of the bed, with which she will lie parallel and on her left side; her left arm will be thrown behind her and can lie over the edge of the bed; her head will be on the pillow, her knees drawn up—the right one rather higher than the other, and the patient's clothing will be pulled out of the way, leaving her with hips uncovered by anything except a sheet which lies over her. You will stand by the doctor, and pass to him the lubricant when he is ready to use it, and such instruments as he calls for.

After examination the patient may or may not require a douche of 1 in 2,000 sublimate lotion, or she may be merely made comfortable in bed again. If an anæsthetic has been administered you will, of course, remain with her till consciousness is quite established. Turn her face to one side, put a towel beneath her chin, and have a basin or soap dish in readiness lest she vomit. She would not be given any food for three or four hours after she had recovered consciousness, and then patients usually find a cup of tea and milk the most agreeable thing to start with. Food for the remainder of the day would be liquid, and unless there are orders to the contrary the ordinary diet would be resumed on the following day.

Where a patient is to be examined at the rooms of the doctor, she will have had directions about the attention to her bowels, and all you will be called upon to do will be to prepare her for examination in the following way.

It will be well for the patient to empty her bladder, which is an essential condition preceding all examinations and operations. You will assist her to remove her corset and waistband, and she will lie on the couch on her back with a light rug thrown over her, leaving exposed the abdomen. You will now call the doctor into the room, and when he has finished his examination of the abdomen you will direct the patient to draw up her right knee. You will draw the clothes away from the buttock, and hand the lubricant and instruments as required.

If the patient is to be examined lying on her side, the knees will be drawn up as before, after she has assumed the same position, and if you are given any instruments to hold, you will be careful to hold them at the precise position desired by the doctor. If it be a Sim's speculum, do not cram it into the patient, but pull it towards you as you stand at her back, so that the blade of the instrument that is inserted may draw back the posterior wall of the vagina and provide plenty of room for the examination. Before passing it to the doctor you will have dipped the back of the blade that is to be used in vaseline or some other lubricant. When the examination is completed you will assist the patient to dress, and carefully scrub and cleanse all the instruments that have been used, or boil them if so directed.

### To Nurses.

In order to increase and vary the interest in the *Mirror*, we invite contributions from any of our readers in the form of either an article, a paragraph, or information, and will pay a minimum of 5s. for each contribution. All rejected manuscripts are returned, and all payments are made at the beginning of each quarter, *i.e.*, January 1st, April 1st, July 1st, and October 1st.

### Pictures from Italy.

### A DISTURBED SICK-ROOM.

THE wind swept down from Fiesole in the bitter gusts which ruffle the pleasures of a Florentine spring. In the huge block of buildings erected just outside the line of railway that has taken the place of the mediæval wall, every door banged and every window rattled. A smith on the ground floor hammered noisily; birds, dogs, and children whistled and howled and cried with as much gusto as if they were denizens of a London alley, and knew nothing of the City of Flowers.

At the third floor a familiar smell of carbolic met one, coming from under a closed door on the left. There was the usual sound of noisy voices within, and for some moments my knock was unheard. When the door at last opened an excited man seized me by both hands and drew me quickly into the passage.

"You have come at last, Signora," he said in rapid Italian. "You have so much kindness for my poor wife. Four weeks she has this fever, and now this other matter—she will not get well, oh, no! but she will be glad to hear your English tongue. Come, she is in here."

He threw open a door and motioned me within. I caught sight of a woman bending over the bed, evidently doing something for the patient, and drew back.

"Has she a nurse with her?"

"Only the obstetrice," he answered. "She will go soon, and you will stay with us, Signora—*non è vero?*"

I got him to take me into a side room and tell me about the condition of things. He had married an English girl with whom I had been slightly acquainted, and a few months after the marriage she had contracted typhoid, which, within the last few days had caused a miscarriage. The case was serious, and there was little or no nursing. The midwife came every morning, and a nun sat up with the invalid at night. The husband said he had not taken his clothes off for a week, and he looked like it. He gave his explanation noisily—every word could be heard through the little flat, and all the time the casements and doors, shaken by the wind, kept up a deafening accompaniment.

I put on an apron while Signor Manetti left me to answer a ring of the bell. A tall, grave man, who turned out to be the obstetrical physician, went into the sick-room followed by the husband. A fat contadina came from the kitchen opposite with an ice-bag, which she gave to the midwife. All four remained in the patient's room. A confused noise of several people talking at once came through the half-closed door; then Manetti sprang out and took me by the arm.

"Come, Signora; the doctor is here; why don't you come?"

The sick-room was very small and crammed with furniture; bottles, glasses, clothes, the accumulation of weeks of neglected illness were standing about, or piled on every available space of table and floor. The four people already in the room stood around the bed all talking except the doctor, who was trying the patient's pulse. I could not see the patient from where I stood, but the face of the medical man was very grave. He took a bottle from the table and measured something into a glass.

"She is very much excited," he said; "I'm going to give her a strong sedative; she must not be disturbed for several hours."

He went away, and I made an ineffectual attempt to get his wishes carried out. The midwife was an amiable fusty person in a black gown and a bonnet which had seen better days—an Italian Mrs. Gamp. She agreed to everything I suggested, but I could not get her out of the room. She said that I had a good face and was very *simpatica*, and that she was very glad that someone was coming to look after the poor signora. She herself had only time for the important things, douching, enemata, &c.; she would still come every morning to attend to these.

The patient was beginning to feel the effect of the sedative, but she seemed to recognise me, and put out her hand. She was in much the same forlorn condition as the room, and her curly hair was tangled and matted under the ice-bag that kept slipping over her eyes.

"It is nice to hear an English voice," she said, faintly, "the Italian bothers me now I am ill. Send them all away and stay with me—I think I could sleep."

I almost pushed the husband and the two women into the passage, where they were still talking when the door bell rang again. A short, fat man with a bald head, evidently the fever doctor, bustled in and came into the sick room; all the others followed. He tried the pulse and asked the midwife some questions. It seemed to strike him for the first time that the patient was dirty and neglected.

"We must wash her," he said, loudly and cheerfully; "we must put her on some clean linen and change the bed—she will find herself more comfortable."

They all set to work with the utmost goodwill to wash the invalid. Another bed was wheeled from a corner and she was lifted on to it. Under other circumstances I should have admired the spirit in which the doctor took off his coat, rolled up his shirt sleeves, and did most of the work himself. At present the proceedings appeared the reverse of sane, and the poor woman's face threatened convulsions. When they commenced I had made a desperate attempt to tell the doctor of the strong sedative that had just been administered, but he refused to understand and evidently regarded me as an interloper. When the tidying up was finished the doctor and midwife seated themselves comfortably on the vacated bed, while the husband sat on the one occupied by the sick woman, holding her hand. The servant fetched a chair for herself, and they talked over every case of illness and death in the neighbourhood, not omitting the funerals. I felt that the case was hopeless, and went home, leaving a note for Signor Manetti.

Early next morning he was at my door, more unshaven and excited than ever; the tears were streaming down his face.

"Why did you go away, Signora?" he said. "She asked for you—she did not sleep at all and she has had convulsions all night. Pray come back with me—she will ask again."

"I can do nothing—you have already too many people there," I said.

"But she wants an English nurse—we will do everything you wish, Signora."

"Signor Manetti," I said, firmly, "if you will go back and send away the midwife and the contadina, leaving only yourself in the flat to help me, I will come."

"I will do everything, Signora."

"Moreover, you must promise not to put foot in your wife's room without my leave."

"Yes, yes, Signora.'

"Very well, I will come in half an hour; but if there is anyone in the flat excepting yourself I shall come away again."

When I arrived at the flat a little later I found he had kept his word. He seemed very tired and depressed, and it is possible that he had found it a difficult task to get rid of the women. He was glad to be sent out of the sick room, for his wife was light-headed, and babbled incessantly. I kept him employed filling ice-bags and taking away various articles that were not needed in the patient's room. The tall, grave doctor had already paid his visit, and when the fat little man came he went through the usual forms quickly and quietly, overcome by the fact that there was no one t talk to. He made up for it, however, by shutting himself in the sitting-room with Manetti for about an hour. I kep the doors closed, and their voices disturbed me little, while the occasional gurgle of a flask of Tuscan wine showed that they were happily employed. Once the voices rose rather loudly, and I went to them at once.

"Dottore, caro mio," I said in my best Italian, "You want the patient to be kept perfectly quiet, do you not? I—as your subordinate—wish to carry out your orders in every respect. Will you, then, as the walls are thin, ask Signor Manetti not to speak so loudly."

"Certainly, certainly, cara Signora," said the doctor amiably, and after that they spoke softly.

During the long weeks that followed I had many a fight with the relatives and other people who called at the flat to make inquiries. They could not understand why they might not see the patient—the Italian sick-room being usually a place for meeting and gossiping. Then they would talk at the front door which was close to that of the sick-room. By some strange freak of Nature, the Italians, while possessing the softest language in the world, are gifted with the most discordant voices.

In other ways the place was kept fairly quiet. Meals were sent in from a restaurant, and kind friends brought supplies of beef-tea and jellies for the invalid. Signor Manetti, once persuaded of the necessity of quiet, went to the other extreme. He wore felt slippers and went on tiptoe. He did not even whisper, but conversed with those expressive gestures, facial and manual, in which his nation is so rich.

At night a little nun with a sedate round face came to relieve me from the sick-room. She knew nothing of nursing, but was used to sitting up with the dying, and was quick to notice any change in the patient's face. She would sit all night telling her beads, only leaving off to fill the ice-bags, or to give nourishment. If anything further was required she called me up, and I was in continual fear of bed-sores from the state in which I usually found the patient's bed. After a few nights I managed to get another English nurse to take night duty, and then I was able to obtain some sorely needed rest.

With the medical men we got on famously when they had once assured themselves that we were willing to carry out their orders. The fat little man was rather afraid that the patient would catch cold under our system of open windows. He also thought it an excess of zeal when we reduced the patient's matted tangle of curls to order, but with these exceptions he looked on our methods with friendly eyes, and later on, with volubly expressed admiration.

### The London School Nurses' Society.

On Monday afternoon Lady Aberdeen presided over the first annual meeting of the London School Nurses' Society. There were present: Lord Reay, Sir Henry Burdett, Mr. Brudenell Carter, Mr. W. C. Bridgeman, Mrs. Ruth Homan, Miss Amy Hughes, &c.

The Honorary Secretary (Miss Honnor Morten) read the letters of regret for non-attendance and the report for the society's first year's work. The report stated that the object was to supply visiting nurses to elementary schools in poor districts on the same lines as the National and Metropolitan Nursing Association. The appointment in January of a Queen's nurse to Southwark makes the third employed by the society. A nurse costs £50 to £60 a year, her full time not being needed, and one nurse can visit from five to ten schools twice weekly.

The Honorary Treasurer (Mr. Bridgeman) presented the financial report, from which it appeared that the income was about £47, and that the expenditure this year was estimated at £123. Fortunately the balance at the bankers—accruing chiefly from an entertainment given last year in the Queen's Hall and from special donations—would, Mr. Bridgeman said, enable the society to meet all liabilities, with a deficit of 2s. only. This, he thought, would prove satisfactory to those who demanded strict economy in the administration of public funds. The society at present employed three nurses. The work in London required ten. Estimating that each nurse cost £50 a year, and her dressings at £4, an income of £540 would be necessary to extend the scheme throughout the metropolis. It was obvious, therefore, that the subscription list must be largely increased.

Lady Aberdeen, in a few words, expressed her pleasure at being asked to take the chair, and her deep interest in the work. She said all mothers—everyone who had to deal with children—knew how much could be done in illness by paying early attention to the little ailments of children.

Mrs. Ruth Homan pointed out that the nurse's instructions to the mothers of the children were as valuable, and often as much appreciated by them, as the actual attention given at the school. A nurse's regular visits not only checked the spread of infection, but once fairly started she could undertake the care of ten instead of five schools. We had not, as in America, organised medical inspection; therefore, we had the greater need for the work of the school nurse.

Mr. Brudenell Carter, F.R.C.S., commended the society's work because of his practical knowledge of the evils it combatted, acquired in his experience as ophthalmic surgeon to great London hospitals. "Dirt and neglect were," he said, "potent factors in causing disease." What was true of the individual was still more true of the crowds in our densely populated districts, and the schools were centres for disseminating disease. The experienced eye of the nurse would detect the first symptoms of disease in any of the children, and they would be placed under proper care. She would also preserve amongst them that great armour of sound health, an unbroken skin. Scrofula was often caused by the invasion of the tubercular bacilli through cracks in the lips and nostrils; erysipelas was carried in the dust; an affection of the eyelids which attacked myriads of children was the product of dirt. It destroyed in time the eyelashes—the natural protection of the eye—and left unsightly, reddened rims that must tell against the sufferer in commercial occupations. The work of the society was calculated to be of the greatest value to parents, children, and the community.

Miss Amy Hughes, in moving the re-election of the committee and hon. officers, said she was able as the first nurse engaged in this work to endorse from personal experience what the previous speakers had stated. She also declared that the moral effect of the nurse's visit was great, it being regarded as a disgrace by the youngsters to be sent up to nurse for dirt.

Sir Sydney Waterlow seconded the motion. He thought such important work ought not to be left to voluntary effort, but that it ought to be provided for by the machinery of the State.

Lord Reay, in proposing a vote of thanks to Lady Aberdeen, said he did not care to see any more sanitary inspectors. One inspector generally condemned what his predecessor had done. He appreciated much better what was done by voluntary effort than what was done by the State.

Sir Henry Burdett, in seconding the vote, expressed regret that such a society should want funds. It was a striking feature, he remarked, "that the schools visited by nurses attracted most children. They were eager to go where their pain was relieved. Considering the service rendered by the nurses in administering to the small miseries of small humanity, and the economies effected in the rates, he thought that Lord Reay, as chairman of the London School Board, might bring before the Board a resolution in favour of a substantial annual subscription to the funds of the society.

### St. John's Hospital, Lewisham.

LAYING THE FOUNDATION-STONE OF THE NEW WING.

The Nursing Sisters of St. John the Divine had the pleasure of seeing the foundation-stone of the wing extension of their hospital on Morden Hill, Lewisham, laid by the Duchess of Albany last week. The large tent erected over the stone was prettily decorated with flowers and bunting of red and white, whilst it was filled to overflowing with the numerous guests assembled to witness the interesting ceremony. The Duchess arrived about five o'clock, and was received by the Bishop of Southwark, the Chaplain, the Sister Superior, and the Treasurer, Mr. Monro. The religious service was begun immediately by the Chaplain. The Bishop blessed the stone, and Canon Rhodes Bristow read the address. The latter was practically a concise history of the community and work of St. John the Divine, which was founded fifty years ago to ensure a high standard of character amongst the nurses and skill in nursing. This aim is achieved by training ladies and respectable women thoroughly under a Superior and Sisters.

Many eminent names appear on the roll of presidents. The first was the late Duke of Cambridge, and amongst others are Earl Nelson, Dr. Todd, of King's College Hospital, Bishop Christopher Wordsworth, and Bishop Walsham How.

The mother home of the sisterhood is at Drayton Gardens, South Kensington, but it possesses working centres at Deptford, Poplar, and Lewisham, as well as a convalescent home at Littlehampton. At the present time there are twenty sisters and eighty-six nurses, probationers and lady pupils.

The sisterhood first began work in the Lewisham premises in 1884 with 28 beds. The pressure on their space is so great that no fewer than 237 patients were admitted last year. The new wing will provide 34 beds, whilst six beds will be retained in the old house. The nursing staff will be accommodated in the old quarters. Mr. Holloway, of Deptford, has undertaken the building contract for the new wing for £5,370. This has been defrayed by special donations amounting to £5,000, and the residue of the munificent gift of £5,000 for the work at Deptford and the neighbourhood.

In conclusion, Canon Bristow said that the contents of the purses presented to Her Royal Highness and the collection taken would be devoted to the furnishing and equipment fund. He gratefully acknowledged the community's indebtedness to the neighbouring medical men and clergy in their name, and thanked the Duchess of Albany for her presence.

A glass urn containing a copy of last year's report, and two of the medals of the community having been deposited under the stone, the architect presented Her Royal Highness with a copper trowel bearing the crest and motto of the Sisterhood in silver. After the needful preparations, she said in a clear voice, "In the faith of Jesus Christ we place this foundation stone in the name of the Father, the Son, and the Holy Ghost."

The Bishop resumed the service, and then about forty small boys and girls presented purses containing from £5 to £25. These with the collection made during the singing of the final hymn realised £367.

The proceedings terminated with the National Anthem.

Tea was provided in the garden, and the Duchess and many of the guests took the opportunity of inspecting the hospital.

### Minor Appointments.

Belgrave Hospital for Children, 79, Gloucester Street, S.W.—Miss M. E. Graves has been appointed Dispenser of this hospital. Miss Graves was trained at the London College of Pharmacy for Ladies, 57, Westbourne Park Road.

### Typical Patients.

THE MEAN PATIENT.

She had a mean, old face, the lips were thin, the nose was pinched, and the eyes were small and suspicious-looking. Even the scanty wisps of grey hair that would straggle on the pillow were ungenerous-looking.

Perhaps her hard life, with its terrible struggle for a livelihood, and its great loneliness, had checked any tendency to open-handedness she may ever have had, and now that she was old and ailing her fault seemed intensified.

When she first arrived her bed happened to be at the end of the ward, but the fact that other people had beds nearer the fire so preyed upon her mind that at the first opportunity she was moved to the desired spot.

She watched every plate when dinners were served, and when for some days she was restricted to fluid diet the expression of her eyes as she watched the other meals was something to marvel at.

It may have been a slight consolation to her that even then, when things seemed at their worst, she achieved a triumph. In the hospital it was customary for the patients to provide their own butter. A new patient arrived just then, and Granny sold her now superfluous butter to her. Uncharitable people said it was margarine which had had its distinguishing wrapper removed, and even the charitable were sure the old lady had made a profit on the sale.

It happened once that one of the other patients was ordered the same medicine as Granny, and before the new bottle came from the dispensary she was given a dose out of Granny's bottle. The old lady was very wroth, and complained bitterly. All reasoning was in vain. She scouted the proposal that she should have a dose of her neighbour's medicine to make up. How could she tell it was the same? To the end she remained convinced that her recovery had been retarded by the loss of that dose!

But one day the attention of the ward was diverted from Granny and her meannesses by the arrival of a little golden-haired laddie who seemed dying of broncho-pneumonia. Many anxious days and nights followed while the little fellow battled for his life, and gradually the whole ward grew interested in the struggle, and even Granny never grudged the attention he received.

One evening when the doctor had been hastily summoned and the end seemed near, Granny heard a request for brandy, and leaning forward she said eagerly, "Give him some of mine, nurse." Here was, indeed, a sign of grace when Granny grew generous, even though it happened to be with hospital property.

But Teddy won his way slowly back to health, and chattered ceaselessly from morning to night. He made friends with Granny, whose bed was next his cot, and she listened to him with whole-hearted admiration; she seemed transformed.

His coming "birfday" was his great subject of conversation, and Granny was as interested as he. "Supposin'," she said one day, "that someone with lots of money," and she dwelt on the words as if they were a charm, "was to let you choose what you would like for your birthday? What would you say?" "A wooden 'oss," answered Teddy, promptly.

That night, as nurse went down the ward, she heard a whisper from Granny's bed. The old lady was fingering a little heap of coppers on her locker. "It's his birthday to-morrow," she said, half shyly; and then, impulsively thrusting the coppers into nurse's hand, she continued, "I want you to get him a wooden 'oss." "Thank you, dear," said nurse, touched by the sacrifice. But as she moved away a thin old hand clutched the curtain, "And as big as you can get for the money," added Granny.

### Echoes from the Outside World.

AN OPEN LETTER TO A HOSPITAL NURSE.

Princess Christian—in whom all nurses are particularly interested—has been working, if possible, harder than ever this week. For three days she has been selling at the Royal School of Art Needlework, assisted by many ladies of the committee. Her stall, instead of being in the crowded vestibule, was arranged in the oak-room, and the consequence was greater comfort, though, of course, the oak-room was never allowed to be in peace for long. Every one who attended the sale of work liked to feel the Princess had personally sold them something, and the smile and "Thank you very much" which was the reward of the purchaser decidedly went some way towards enhancing the value of the article selected. A special feature of the work shown was the judicious introduction of embroidery and rich brocade into substantial articles, made of wood. Small revolving bookstands had the top and panels filled in with fine embroidery, diminutive chests of drawers were upholstered in Japanese brocade, and letter racks, made of oak, had the ends adorned with needlework. I must not forget to tell you about the tea-room. It was so delightful that I felt I could sit there all the afternoon if I had not known so many thirsty souls were waiting for my seat. The walls were covered with white fluted muslin, with big bunches of pink and white pæonies, apparently tied on the curtains by loopings of pink satin ribbon. The effect was so cool and pretty, and the roof of crossing trails of ivy and asparagus-fern heightened the feeling of rest and refreshment which gratified the visitor even before the tea was partaken of. I could not help wondering whether the new buildings—of which the Prince of Wales lays the foundation-stone on Friday—will not probably be on too large a scale to admit of such unique treatment. So that it was with feelings of regret, as if parting from an old friend, that I looked behind me as I passed out of the door.

---

Even if you are not fond of cricket you must have heard echoes of the exciting contest at Lords between England and Australia, in which the Australian bowlers almost carried all before them. For, whatever may be said about the match, there is no doubt that the lightning bowling of Jones in the first innings was the principal factor in securing the victory. It beat the Englishmen so thoroughly that, as the experts say, they were "practically nowhere afterwards." But MacLaren made us all feel burning with enthusiasm at the cool, plucky way he played in his second innings. A man sitting beside me was too much absorbed in the game to talk, but he had just three adjectives which he kept murmuring under his breath at intervals, "Magnificent!" "Superb!" "Wonderful!" The regularity with which he gave a turn to these exclamations of delight would have made me laugh if I had not been so interested myself watching the steady, and yet brilliant, play that I could think of nothing else. It was all the nicer for MacLaren because, when he was included in the eleven chosen to play against Australia, lots of folks declared it was lunacy to put him in a representative team owing to his not having played in a first-class match. As it turned out, it would have been lunacy to have left him out, for he did more than anyone else to retrieve the fortunes of the day, played like a "crack," made 88 runs, and carried his bat. In company with many hundreds of other ladies, we took our lunch with us, and made a sort of picnic, in order to avoid losing our seats by moving away during the interval, and we were exceedingly glad that we had. Those who depended on the refreshment buffets were not nearly so lucky, for the attendance was so huge that some visitors had to be grateful for a crust of bread, I was told.

Speaking of cricket, there is such a strange mug in the Crockery Exhibition now being held at the Bethnal Green Museum. It has been made to keep alive the memory of a grand match played at Lord's Ground, Marylebone, between the Earls of Winchester and Darnley for 1,000 guineas. The event happened more than a hundred years ago, and it is specially interesting to recall it just at the present time. Those nurses who live anywhere near the exhibition would enjoy an hour there. The jugs and mugs, plates and bowls, form a most unique assortment, and are lent by Mr. Henry Willett, of Brighton, who collected them "with a view to develop the idea that the history of the country may, to a large extent, be traced on its homely pottery," most of which comes from Staffordshire and the old potteries of the North and West. The various events depicted make one rub up one's history, too apt to get rusty with little time for study. There are portraits of Charles I., Charles II., Cromwell, William and Mary, Anne, and George I. and II. Also of the young Chevalier, and the Duke of York, who marched up a hill with his 10,000 men and did nothing more except bring them down again. Several jugs and mugs allude to the time when England lived in terror of the Napoleonic invasion, many sing the praises of Nelson, and murderers, highwaymen, and pickpockets are all commemorated. But the manufacturers of the criminal pictures have kindly added a few words of letterpress in most cases, lest the horrors they committed should have been forgotten.

---

An excellent little society has been formed lately to help those whose lives are darkened from want of sight. It is called the Fellowship of the Blind and Seeing, and it originated with a blind lady, whose affliction made her think of the numbers who have to spend long dreary evenings at home, wishing that they had been asked to some of the entertainments of which they had hear their friends frequently speak. The associates consist of the cultured blind and certain hostesses, with kindly, sympathetic hearts, who promise, when possible, to include a few of the blind members amongst those to whom they send out invitations for a musical or social evening. Surely, many who entertain largely will be glad to enrol themselves in this new Fellowship, and thus to feel that their party, which perhaps is a bore rather than anything else to half the guests, can be a distinct source of gratification and delight to some man or woman of good social status who is debarred from so much which makes the joy of the world to those who have eyes to see.

---

I was calling at a house the other day, when a lady was shown in, attended by her little son about three years of age. I noticed that the child, who was a fine big boy, walked rather strangely, and when he sat down I was surprised to find that he wore neither shoes nor socks, but the queerest little sandals, which were made of stout pieces of leather, curled up at the end like Turkish slippers, and fastened with buckled straps. One went across the instep of the foot, and the other round the ankle. The little toes all showed distinctly, but as it was not a dirty day they were only dusty, not really soiled. In wet, muddy weather the effect would be anything but nice. I was told that the child had not even worn sandals till he was turned two, and that he thought the promotion to any kind of footgear was very grand. He was a very modern young man altogether, for as well as having been brought up on strictly hygienic lines, he had been taught to speak nothing but French, because, his mother explained, that if he was good at foreign languages he could pass his examinations into the Army so much easier! Arranging for Army exams. before your child has lived three summers seems a little premature. In this case too, the young person looked as if he would have a strong will of his own some day, and might object to have his profession chosen so early for him. Such a possibility had evidently never entered his mother's head!

### A Book and its Story.

MRS. OLIPHANT.

The impression left upon the mind by Mrs. Oliphant's "Autobiography and Letters"* is that she herself, in spite of the loss of all that was dear to her, and the never-ceasing strain which the necessity of making an income for the support of her family entailed, was not an unhappy woman. She had a natural gift for writing, which in itself was a source of unfailing pleasure to herself throughout life. It came, she said, "as easily as breathing or speaking." Her books were her work, and a natural occupation, "only they were never so good as I intended them to be." Comparing her circumstances with those of George Eliot, she cries, "How I have been handicapped in life! Should I have done better work if I had been kept, like her, in a mental greenhouse and taken care of?" But Mrs. Oliphant's was a nature that would never have sunned itself in isolated luxury of this sort. From her childhood her thoughts and heart were set upon serving and helping others. She had never been able to think of herself. "I have always had to think of other people, and to plan everything—for my own pleasure, it is true, very often—but always in subjection to the necessity which bound me to them." Fortunately she was blessed with perfect health, which never, until the very end, failed her in any way. She had, too, a peculiar buoyancy of spirit, sustained, undoubtedly, by firm faith and trust in the love and wisdom of her Heavenly Father. In 1852, when in her twenty-second year, she married her cousin, Frank Oliphant, who was an artist and designer of stained glass. Seven years later he died in Rome, leaving his young widow almost penniless. She had then two little boys, and her infant daughter was born after her husband's death. As soon as it was practicable Mrs. Oliphant came to England, and from thence went to Edinburgh. Her literary efforts at this time were, naturally, not very successful, and her staunch friends and publishers, Blackwood Brothers, rejected several articles. She, nothing daunted, feeling that she had the power to do something more worthy of herself, and goaded by the fact of being already in their debt for money advanced on future contributions during her husband's illness, decided to write a serial novel for their magazine, a proposal the firm were not inclined to entertain. "They shook their heads, and did not think it would be possible to take such a story, both very kind and truly sorry for me, I have no doubt. I think I see them now against the light standing up—John with his shoulders hunched up, the Major with his soldierly air, and myself, all blackness and whiteness in my widow's dress, taking leave of them as if it did not matter, and, oh! so much afraid they should see the tears in my eyes."

Calamity at no time seems to have been able to crush Mrs. Oliphant's spirit; it seems rather to have acted as a stimulant, and she realised that action, and that an immediate one, was the only alternative at this critical moment. Returning home, she sat up all night "in a passion of composition, stirred to the very bottom of my mind." The result was the first number of the very successful "Chronicles of Carlingford," which appeared at once in "Blackwood." "The Chronicles," like so many of her best and more recent works on biography, history, and fiction, &c., were unsigned, and much conjecture was raised at the time as to their authorship. The *Saturday Review* put them down to G. Eliot, and announced emphatically "that they could have been written by no one else," which the real author considered a high compliment to herself, as it undoubtedly was.

From this time Mrs. Oliphant went successfully ahead. Taken together, her novels, we are told, "can hardly number less than a hundred, but these for a long time back were by no means her works of predilection." Besides several well-known biographies, some involving great labour and research, "there are the brilliant papers on George II. and Queen Anne," "A Child's History of Scotland," and the "Literary History of England," "The Makers of Venice, Rome, and Jerusalem." Much labour was involved in the composition of these works, all of which, "with the exception of 'Rome,' having been written from materials collected on the spot." Her last writings, "one of the most wonderful sets of writing in our language, began very simply and sweetly with 'A Little Pilgrim,' and went on through various 'stories of the seen and the unseen,' reaching a strange poetic power in a beleaguered city and finding . . . a most fitting close in 'The Land of Suspense.'" For fifty years she wrote in all these varied fields of literature, and never, with the single exception alluded to, in the early days of her widowhood, failing to have her writings accepted.

Her letters and life, on the whole, are singularly free from reminiscences of the notabilities, literary and otherwise, with whom she was contemporary. The Carlyles were intimate and valued friends, and there is a touching anecdote of Carlyle himself, who, when hearing of the sudden illness of her son, made his wife write at once to Mrs. Oliphant to allay her anxiety, and say that his sister once had such an attack, which was never repeated. "God bless them,' says Mrs. Oliphant, "that much maligned and misunderstood pair!" and he, she thought, very unlike "the old ogre his false friends made him out to be." Carlyle's dyspepsia may have been responsible for much, but it had also a saving clause from Mrs. Carlyle's point of view. She had paid Mrs. Oliphant a visit, and "was as amusing as ever with anecdotes throwing light upon the domestic relations of the distinguished people of the period." Mrs. Oliphant, impressed by the eccentricity of these relations, "could not but say that Carlyle seemed the only virtuous philosopher we had left." "My dear," replied Mrs. Carlyle, "if Mr. Carlyle's digestion had been stronger, there's no saying what he might have been!"

A little picture of her at home, in the brief preface which precedes the autobiography, brings before the reader vividly her gentle, womanly personality, always evident, but never more strikingly so than in her last illness. "There was always a pleasant welcome and talk of the very best to be found in her modest drawing-room. If visitors were congenial, her charm of manner awoke, her simple fitness of speech clothed every subject with life and grace, her beautiful eyes shone (they never sparkled—a notable distinction), and the spell of her exquisite womanliness made a charmed circle around her."

Writing to Dr. Boyd, A.K.H.B., towards the close of her life, when her cup of sorrow was welling up to the brim, she says: "The one good thing I am conscious of is the increased tolerance—nay, enjoyment of the loneliness which is inevitable, and the great calm, all-sustaining sense of a Divine Unseen, a silent companion, God walking in the cool of the garden, which, after all is best." And again, in her last hours, when all her loved ones had been taken from her, and she was left alone. "God is very good, He gives me everything." He had given her, without doubt, the ornament of a meek and quiet spirit, which enabled her to bear with fortitude the trials by which her life had been beset.

We must bring to a close our notice of a very interesting autobiography, whose not least interesting parts are those recording Mrs. Oliphant's impressions of Rome, Venice, Florence, and the Holy Land, written on the spot with the delightful lucidity and veracity in description of which she was mistress.

* "Mrs. Oliphant's Autobiography and Letters." Edited by Mrs. H. Coghill. (Blackwood, Publishers. 1 vol.)

### Bazaar in Aid of the Charing Cross Hospital Special Appeal Fund.

The Princess Louise, Marchioness of Lorne, opened the grand bazaar at the Albert Hall on Wednesday, and subsequently herself presided over the hospital stall. A feature of this stall was some Kaiser silver plate from the Duke of Saxe-Coburg and Gotha, the president, and embroideries and printed work from his daughters. Among other Royal givers to the stalls were the German Empress and King Leopold; while the Queen, who has been patron of Charing Cross Hospital since 1833—when the Duchess of Kent gave permission that one of the wards should be called the Victoria, after the young Princess—sent a cheque for £250 as a contribution. It was a remarkable feature of the bazaar that at the last moment the stalls had to be slightly enlarged, owing to the generous assistance given by all sorts of people to the committee, with Mrs. Arthur Paget, the honorary secretary, at their head. Mrs. Paget, in addition to all her duties, had charge of the most striking stall, which took the shape of a Greek temple. Greece, however, was only one of many nations represented. France, with French fruits, French bon-bons, and French hats; Denmark, with Danish butter, Copenhagen porcelain, and Danish cherry brandy; Japan, with a mass of antique tapestry and embroideries; Egypt, with gay-coloured rugs; and, of course, England, Germany, Russia, and India were strongly in evidence. "South Africa," with its roof crowned with palms, shields, and assegais, and covered with lion skins, was also conspicuous; and at the Kronthal American bar, where Transatlantic beverages were dispensed by aristocratic ladies in lovely frocks, a roaring business was done. The refreshments were supplied by Messrs. Gunter for the benefit of the hospital, and Miss Wilson Seager presented the flowers and plants. In fact, everyone was so liberal that the result realised ought to be very satisfactory indeed. The concert and dramatic performances, in which numerous celebrities took part, and the sale of souvenirs must enormously tend to swell the total received at the doors.

### Appointments.

Steyning Union Workhouse.—Miss A. M. Sharrock has been appointed Superintendent Nurse of the sick wards. She was trained at Birmingham Workhouse Infirmary, where she was sister for four years; she has been for eighteen months night superintendent of the Hahnemann Hospital, Liverpool; first assistant matron and superintendent at Crumpsall Infirmary, Manchester, from July, 1897, to October, 1898; and subsequently superintendent nurse at Romford Union Infirmary.

National Hospital for the Paralysed and Epileptic, Queen's Square, W.C.—On June 13th Miss Eleanor Caroline Vernet was appointed Matron. She was trained at the Middlesex Hospital and at the hospital to which she has now been appointed; at the latter she has been staff nurse, night superintendent, and day sister. Miss Vernet was at one time sister of the male surgical wards at the Middlesex Hospital.

Caterham Asylum.—Miss Jeannie MacLeod entered upon her duties as Superintendent Nurse on June 14th. She was trained and afterwards staff nurse at the Royal Infirmary, Aberdeen. She received her training in mental nursing at Larbert Asylum. Miss MacLeod has also been district nurse at Sowerby Bridge, Yorks.

Mill Road Infirmary, Liverpool.—On June 14th Miss Ellen Bracewell was appointed Matron. She was trained at St. Bartholomew's Hospital, Rochester; and has been head nurse and assistant matron at the infirmary, East Dulwich Grove, London, S.E.

Tottenham Hospital.—Miss Margaret Fox has been appointed Superintendent of Nurses. She was trained and was afterwards ward sister at Guy's Hospital, and she has been ward sister at Sunderland Infirmary.

Brighton and Hove Hospital for Women.—Miss E. J. Cartwright has been appointed Matron. She was trained at St. Bartholomew's, and she has since been matron of the New Hospital for Women, Euston Road, &c.

### Presentations.

Shoreham Workhouse.—Miss Mowat, late superintendent nurse at the infirmary, has been presented with a handsome carved-oak inkstand on behalf of the medical and nursing staffs on the occasion of her leaving to occupy the position of superintendent nurse at the Birkenhead Union. During the six years she has been at Shoreham Miss Mowat has proved herself thoroughly efficient and tactful, and has won the very hearty appreciation of all with whom she has come into contact. It has been mainly through her instrumentality that the infirmary accommodation has been brought to its present state of perfection. The presentation was made by Mr. M. E. Dovaston, chairman of the Infirmary Committee. The following address was presented to Miss Mowat by the patients: "To Sister Mowat,—We, the patients in the infirmaries of the Steyning Union, in offering for your acceptance this humble tribute of our affection and esteem, desire to express our heartfelt thankfulness for the many benefits that have accrued to us under your wise and kindly rule during the past six years. It is no idle form of words to say that many of us will feel very deeply the severance of the tie that has united us so long, and our prayers and best wishes will follow you that your health may be preserved and all comfort and happiness attend you in your new sphere of duty and usefulness. Shoreham, June 10th, 1899."

Brighton and Hove Lying-in Institution, Hospital, and Dispensary.—On the occasion of Miss Parry leaving this institution she was presented by the staff at West Street with a very pretty silver cream jug, sugar basin, and tongs; and by the superintendent and private staff at 9, Portland Road, Hove, with a very handsome set of silver-mounted brushes and combs, with monogram engraved on each, as a tribute of love and esteem in which she is held. Miss Parry who was also the recipient of other gifts, has filled the office of matron of the institution for over three years, and she leaves it taking with her the good wishes of all with whom she has come in contact.

Hospital for Women, Soho Square.—On Tuesday Sister Agnes, who has accepted a responsible position at the University Hospital, was presented with a silver tea service in recognition of her many years' faithful discharge of her duties as sister-in-charge of the theatre. The presentation was made in the presence of Drs. Carter, Smith, Holland, Mansell, Moullin, and Oliver, and several suitable and congratulatory speeches were delivered.

### National Hospital for the Paralysed and Epileptic.

### THE MATRON'S RESIGNATION.

We have received the following from the hospital authorities:—"The resignation by Miss Rachel F. Tweed of the post of lady superintendent is a source of great regret to the management, who have recorded their sense of her valuable services. Considerations of health compel Miss Tweed to have recourse to a less laborious sphere of work, and in conjunction with another lady she is, therefore, about to open a nursing home. Miss Tweed's connection with the hospital extends over ten years, and during the last three years she has held the responsible post she is now relinquishing. Under her supervision important improvements have been introduced into the nursing and general domestic arrangements, and she leaves everything in a condition of full efficiency. By the able and conscientious discharge of her duties Miss Tweed has commanded the esteem of all, and in not less degree have her high personal qualities won for her the regard and affection of those with whom she has been more intimately associated."

### RUSSIAN FAMINE RELIEF FUND.

Mr. J. T. Woolrych Perowne desires to acknowledge the further sum of £4 6s. received on behalf of this fund.

### Everybody's Opinion.

[Correspondence on all subjects is invited, but we cannot in any way be responsible for the opinions expressed by our correspondents. No communication can be entertained if the name and address of the correspondent is not given, as a guarantee of good faith but not necessarily for publication, or unless one side of the paper only is written on.]

### NURSES AND THE WORKHOUSE PORTER.

Henrietta Hannath, Superintendent Nurse, writes from Union Workhouse Hospital, Eastville, Bristol: In reference to your leaderette with regard to the resignations at Eastville Workhouse, I beg to state that an entry of the outgoing and incoming of the hospital staff has always been kept by the superintendent nurse in compliance with the regulations stated in the hospital porter's book. The resignations were tendered after a resolution of the Guardians that for the future nurses were to take a pass to the workhouse entrance, though the hospital is away from the house. On learning the Poor Law order *re* the porter's lodge, the resignations were withdrawn. The Board of Guardians has decided to build a lodge at the entrance gates of the grounds, which everyone will of necessity pass, and in consequence be entered in one book. Of the courtesy and tact of Mr. Wethered, the Government inspector, I cannot speak too highly.

### A READING SOCIETY FOR NURSES.

"L. G. M." writes: "A Nurse" asks in The Hospital of June 10th whether a reading society could be formed to help nurses who are out of touch with hospital and with up-to-date methods and work. It has struck me that such a society could be easily formed if a sufficient number of nurses could be found to join it. With a view to its formation, I have drawn up a set of rules for such a reading society. The proposed rules are subject to hints and suggestions from others, and I should welcome any such hints. I should propose, in selecting the books to be read, to choose those which would not be difficult for nurses to obtain, and I should make the examination papers short ones. The papers should be corrected and returned to the writers. I append a list of rules "on approval," and any suggestions or ideas upon the subject would be, as I say, more than welcome. All letters containing a stamped envelope for reply will be answered immediately.

Proposed Rules.

1. One book dealing with medical subjects will be suggested at the beginning of the month, to be read by members during the month.
2. A short examination paper will be set upon the book read at the end of each month. Answers to the paper to be sent in before the end of the month following.
3. Once a quarter, if wished, a subject will be given upon which short papers may be written by members. The subject will deal always with practical nursing matters.
4. Subscription for nurses, 2s. 6d. yearly.
5. All communications to be addressed to Miss Moberly, 14, Portland Place, S.W. A stamped envelope must in all cases be enclosed if a reply is required.

### TALKING "SHOP."

Alice Fitzgerald writes: Would you have the kindness to allow me to draw the attention of your readers to the grave temptation besetting young nurses of talking "shop"? By "shop" I mean the "cases," the ins and outs thereof, and the matrons and superiors in general. Perhaps this state of things is more marked in nursing and convalescent homes, where, after a short probation time, the young nurse, freshly instructed, is sent out to take charge of a "case"—the very essence of enjoyment to her professional imagination! Even if she does find the thick "bread" of real hard work hugely disproportionate to the delicious "butter" of actual nursing involved, still she will make up for it in some way or another. And, unfortunately, one of these ways is to allow herself to talk and chatter in an unguarded way not only to her patient but of her patient. A public conveyance and the presence of men is no obstacle to this enthusiastic "case-lover." I believe if matrons only knew one half of what goes on between their nurses in a sort of undercurrent they would be quite horrified. For my part, no inducement would compel me to have a young nurse, supposing I were ill. Not because I have any objection to young people, but because the idea of having my ailments lightly discussed with anyone and everyone, in and out of season, even under the graceful guise of a "case," is to me so disgusting and repugnant. It is not only a breach of womanly reserve; it is, according to my view, a disgraceful disregard of the commonest rules of modesty.

### Examination Questions for Nurses.

This month none of the candidates' papers are sufficiently good to receive a prize. I fear this will be a disappointment, but the object of these questions is to encourage nurses to be thoroughly practical in their work and to use their common sense, which is, alas, the rarest of all senses; and if we allow ourselves to be content with indifferent answers to simple questions on the right solution of which the comfort of the sick so greatly depends, our standard will be lowered and the value of these papers greatly diminished. With regard to the choice of a bed, the question says "speaking generally," which means that you are to consider the question of ordinary illnesses, and does not mean that information is required as to fracture beds. Again, it is not desired that an exhaustive account should be given of the bed clothes. One candidate tells me that the sheets should be of linen or calico; I should be interested in knowing of what other materials they are usually made. Very few lay sufficient stress upon the important question of arrangement of light for the patient's comfort and the convenience of the doctor and nurse. As to ventilation there is much difference of opinion, and some of the answers show a lamentable want of thought. During the autumn this question will be repeated, and I hope with much better results.

With regard to the second question, why a room is chosen at the top of the house for an infectious case, the answers show that the true reason is not understood. Only two candidates have answered rightly—namely, that cool air entering from below drives a current upwards through the house, and would carry infection with it if present in the lower rooms. The answer given by several that infectious germs fly upwards of themselves is quite incorrect; they are equally inclined to settle on the floor, but when an upward current of cool air sweeps through a house, displacing the warmer, it naturally carries with it anything that can be dislodged.

Nurse Despard has not sent in her choice of a prize.

A request has been received for one more question for July; it is a simple one, but I hope all candidates will give their best attention to sending a practical common-sense answer. We do not want fine language more suited to a doctor or a chemist, but we desire to be assured that our nurses bring to their work accuracy, deftness, and, above all, common sense. The Setter of the Papers.

Question for July.

Describe first what arrangements you would make to insure the efficiency of a simple linseed poultice for the chest or limbs made on linen. Secondly, what other groundwork you would use failing linen. Thirdly, how you would make mustard and charcoal poultices. Fourthly, describe a jacket poultice and how you should apply it.

### A New Appointment.

Miss Alice Ravenhill has been appointed Instructor in Hygiene to the Yorkshire Ladies' Council of Education and the West Riding County Council. This is a new post, and has been established by the authorities in question with the object both of promoting public health and of providing efficient training for women wishing to qualify for factory or sanitary inspectorships or as health lecturers. Miss Ravenhill will lecture to these students, and her duties will also include a course of instruction on practical hygiene for teachers and introductory lectures on health subjects throughout Yorkshire. It is hoped that general interest will thus be aroused in matters hygienic, and the value of domestic sanitation more widely impressed upon all classes of the community.

### For Reading to the Sick.

ST. JOHN BAPTIST'S DAY.

THIS is He of whom it is written, Behold, I send My messenger before Thy face, which shall prepare Thy way before Thee.— *St. Matthew* xi. 10. (Gospel.)

"Repent ye, for the kingdom of heaven is at hand."

Lo! from the desert homes,
Where he hath hid so long,
The new Elias comes,
In sternest wisdom strong;
The voice that cries
Of Christ from high
And judgment nigh
From opening skies.

Your God e'en now doth stand
At heaven's opening door;
His fan is in His hand,
And He will purge His floor;
The wheat He claims
And with Him stows,
The chaff He throws
To quenchless flames.

John's note was still, repentance! the axe to the root, the fan to the floor, the chaff to the fire! As his raiment was rough, so was his tongue; and if his food was wild honey, his speech was stinging locusts. Thus must the way be made for Christ in every heart. Plausibility is not fit preface to regeneration. If the heart of man had continued upright, God might have been entertained without contradiction; but now violence must be offered to our corruption, ere we can have room for grace. If the great way-maker do not cast down hills and raise up valleys in the bosoms of men, there is no passage for Christ. Never will Christ come into that soul where the herald of repentance hath not been before Him.

What a favour then is it that God hath reserved us to these better days of His Gospel; wherein the helps of salvation are more clear, obvious, effectual; wherein, as the glory of the latter house exceeded the former, so the means of that incomprehensible glory of the house not made with hands, eternal in the heavens, lie more open unto us! What should we do but both gladly use and sweetly enjoy this unspeakable blessing, which God hath kept in store for us, and walk worthy of so incomparable a mercy? The old Jews lived in the dawning of the day; wherein they had but a glimmering of that sun which would rise: we live after the high noon of that happy day. If we walk not answerable to so great a light what can we look for but utter darkness?—*Bishop Hall.*

Ye haughty mountains, bow
Your sky-aspiring heads;
Ye valleys, hiding low,
Lift up your gentle meads;
Make His way plain
Your King before,
For evermore
He comes to reign.

May thy dread voice around,
Thou harbinger of Light,
On our dull ears still sound,
Lest here we sleep in night,
Till judgment come,
And on our path
Shall burst the wrath,
And deathless doom.

O God, with love's sweet might,
Who dost anoint and arm
Christ's soldier for the fight
With grace that shields from harm,
Thrice Blessed Three,
Heav'n's endless days
Shall sing Thy praise
Eternally. Amen.

### Notes and Queries.

**The contents of the Editor's Letter-box have now reached such unwieldy proportions that it has become necessary to establish a hard and fast rule regarding Answers to Correspondents. In future, all questions requiring replies will continue to be answered in this column without any fee. If an answer is required by letter, a fee of half-a-crown must be enclosed with the note containing the enquiry. We are always pleased to help our numerous correspondents to the fullest extent, and we can trust them to sympathise in the overwhelming amount of writing which makes the new rules a necessity.**

**Every communication must be accompanied by the writer's name and address, otherwise it will receive no attention.**

*Skin—Ventilations—Competition.*

(111) I should be very grateful if you answer me the following questions: 1. Can you recommend me a remedy for excessive perspiration of head and chiefly face? 2. How can I cool a room which is immediately under the roof and gets all the afternoon sun? 3. Are the competition questions in the "Mirror" open to any who are not trained nurses?—*M. N. O.*

1. Consult a medical man; your general health evidently demands attention. 2. Outside blinds are essential. If you cannot afford them open the window top and bottom, and draw down the inside blind outside the window. This prevents the glass getting hot. Window boxes kept well wetted help, as they keep the window sills cool. Get a large block of ice, wrap it in flannel, tie it up in a net, and put a basin under it to catch the drippings. 3. The competitions in the "Mirror" are for trained nurses or probationers.

*Chancery.*

(112) I am a Queen's nurse, and would be very glad if you could give me some information concerning the following: There is an estate in Scotland which belongs to my father, and it is in Chancery, and we have not the means to employ a lawyer. I thought once of writing the Queen on the subject. Would this be advisable? If not, what would be the best thing to do? If you could enlighten me I shall be very thankful.—*A. C.*

Collect all your evidence, put it in proper order, with copies of certificates of births, marriages, and deaths, and draw up the most concise statement possible, then take it to a thoroughly respectable lawyer. Tell him the circumstances of your case, and that you have but little means at your disposal. He will inform you what his fee would be for investigating the matter, and should his verdict be a favourable one he would probably make some arrangement by which he would be paid for his work after you have obtained possession of the estate. Have any arrangement put into writing and stamped. Do not write to the Queen. She does not interfere in such cases.

*Chorea.*

(113) I should be much obliged if you could tell me of a home where a boy (15) suffering from chorea could be received. When under treatment and getting proper food he recovers, but as soon as he returns home becomes as bad as ever. Then, of course, he can do no work, and his parents are not in a position to support him. I should be very glad to hear of a home or school where he could be properly treated, as it is only possible to keep him for a limited period in hospital.—*Nurse Edith.*

The case is a difficult one, and you give very little detail. There are no hospitals or institutions for cases of mere nervous debility. As the patient recovers away from home, would it not be possible to arrange with some convalescent home or cottage hospital for his remaining to do light work in the garden or house after he is well until his strength is fully established?

*Monthly Nursing.*

(114) Can you tell me of an institute or home in the East of London where a lady would gain experience in monthly nursing? I would give few hours daily or take night work.—*A. B.*

Perhaps you could come to some arrangement with the Lady Superintendent of the East-end Mothers' Home, 396, Commercial Road, London, E.

*Home for Epileptics.*

(115) Will you kindly tell me if there is a home in Devonshire for patients suffering from epilepsy, and their fees?—*Nurse Brooks.*

There are no charitable homes for epileptics in Devonshire. To hear of a suitable private one you should either advertise or consult the advertisements in the medical journals.

*Cures for Inebriates.*

(116) A lady who has been very intemperate in her habits for some time past has come under my especial care. She now earnestly desires to overcome her unfortunate weakness, but the craving for drink will, however, occasionally present itself. She persistently refuses to enter any home for inebriates. Is there any other remedy which could be suggested when these fits of intemperance occur, and is there any reason to hope that with strict adherence to certain prescribed rules a permanent cure might be effected.—*Nurse C.*

These are cases in regard to which it is very difficult to give a prognosis. Some, perhaps a third, get well; the rest don't, and that is all one can say. What has to be remembered, however, is that many women in whom the craving recurs more or less periodically are really ill, suffering from recurring maladies, mostly of a neurotic type, for the discomforts, the sinkings, the depression, and sometimes the pain of which they have unfortunately discovered a remedy in alcohol. One must not be in a hurry to throw stones at women for drinking until one is assured that they are not suffering from disease. Even so, however, much may be done by total abstinence to keep at bay the craving, and to render it difficult to get at that "first drop" which, when the craving is on, leads inevitably to an outbreak. We are very sceptical about all medicinal "drink cures," although treatment may, of course, relieve the disease that leads to the desire. The best one can do is to secure healthy and constant occupation, involving a good deal of exercise in the open air, and if one has a choice of residence and can discover a place where odd drinks are difficult to obtain so much the better. Many a lady who would not hesitate to take a glass of sherry at a confectioner's or at a refreshment room would hesitate a great deal about going into a public house. The whole treatment of dipsomania in women may be summed up in first curing the maladies which lead them to drink, and then keeping them out of temptation.

